# HIGD2A silencing impairs hepatocellular carcinoma growth via inhibiting mitochondrial function and the MAPK/ERK pathway

**DOI:** 10.1186/s12967-023-04105-7

**Published:** 2023-04-12

**Authors:** Kuiyuan Huang, Ziying Liu, Zhanglian Xie, Xiaoran Li, Haixing Zhang, Yu Chen, Yiran Wang, Zimo Lin, Chuanjiang Li, Hongyan Liu, Xiaoyong Zhang

**Affiliations:** 1 State Key Laboratory of Organ Failure Research, Guangdong Provincial Key Laboratory of Viral Hepatitis Research, Department of Infectious Diseases, Nanfang Hospital, Southern Medical University, Guangzhou, China; 2 Division of Hepatobiliopancreatic Surgery, Department of General Surgery, Nanfang Hospital, Southern Medical University, Guangzhou, China

**Keywords:** Hepatocellular carcinoma, HIGD2A, Mitochondria, ATP, The MAPK/ERK pathway

## Abstract

**Background:**

The Hypoxia inducible gene domain family member 2A (HIGD2A) protein is indispensable for the assembly of the mitochondrial respiratory supercomplex, which has been implicated in cell proliferation and cell survival under hypoxic conditions. Because the liver has a naturally low oxygen microenvironment, the role of HIGD2A in the development of hepatocellular carcinoma (HCC) remains largely unknown.

**Methods:**

Gene expression data and clinical information were obtained from multiple public databases. A lentivirus-mediated gene knockdown approach was conducted to explore the function and mechanism of HIGD2A activity in HCC cells*. *In vivo and in vitro assays were performed to investigate the biological roles of HIGD2A.

**Results:**

HIGD2A was overexpressed in HCC tissues and cell lines and was associated with a worse prognosis. Silencing HIGD2A expression significantly attenuated cell proliferation and migration, caused S-phase cell cycle arrest, and decreased tumor formation in nude mice. Mechanistically, HIGD2A depletion greatly decreased cellular ATP levels by disrupting mitochondrial ATP production. Moreover, HIGD2A knockdown cells displayed impaired mitochondrial function, such as mitochondrial fusion, increased expression of the mitochondrial stress response protein, and decreased oxygen consumption. Furthermore, knockdown of HIGD2A markedly attenuated the activation of the MAPK/ERK pathway.

**Conclusions:**

HIGD2A promoted liver cancer cell growth by fueling mitochondrial ATP synthesis and activating the MAPK/ERK pathway, suggested that targeting HIGD2A may represent a new strategy for HCC therapy.

**Supplementary Information:**

The online version contains supplementary material available at 10.1186/s12967-023-04105-7.

## Introduction

Hepatocellular carcinoma (HCC) is one of the most common types of primary liver cancer and is the third leading cause of cancer-related deaths worldwide [1]. The 5-year relative survival rate for patients with HCC is only 18%, indicating the poor prognosis of HCC. Patients with HCC tend to be diagnosed in advanced stages due to lack of specific symptoms in the early stage, which probably contributes to poor survival [2]. Despite the development of numerous therapies, including surgical resection, liver transplantation, chemotherapy, molecular targeted therapy and immune-checkpoint inhibitor therapy, HCC remains one of the most difficult cancers to treat [3]. Therefore, there is an urgent medical need to further elucidate the biological mechanisms underlying the development of HCC to explore novel therapeutic strategies.

Cellular mitochondria are critical for multiple cellular functions, including cell respiration, biosynthetic metabolism, energy production, calcium homoeostasis, immune defense, and apoptosis [4–6]. Interestingly, considerable evidence has supported the role of the mitochondria in HCC tumorigenesis and progression [7]. Mitochondrial dysregulation, such as dysregulated adenosine triphosphate (ATP), altered mitochondrial morphology and structure, and increased generation of mitochondrial reactive oxygen species (ROS), has been associated with the occurrence and development of HCC. For example, ATP synthesis promoted by the SIRT1/PGC-1α axis is an important contributing factor to HCC invasion and metastasis [8]. Mitochondria exist as a dynamic network that undergo constant balanced fission and fusion processes, which are important for cellular adaptation to the altered metabolic needs and environmental changes [9, 10]. Nonetheless, when the homeostasis of the mitochondrial network is disrupted in liver cancer, the mitochondria become small spheres or short tubules, and these morphological changes are intimately linked to more advanced tumor stages, metastatic potential, and poorer outcomes [11–14]. Moreover, mitochondrial fission-mediated metabolic reprogramming facilitates the maintenance of stemness of HCC tumor cells by reducing ROS production [15]. Several studies have reported the therapeutic efficacy of targeting mitochondria in liver cancer [16–18].

Hypoxia inducible gene domain family member 2A (HIGD2A) is a small protein of 106 amino acids embedded in the inner membrane of mitochondria and can be induced by exposure to hypoxia and low glucose [19, 20]. HIGD2A plays a key regulatory role in mitochondrial homeostasis. It is required for the assembly of the cytochrome C oxidase (COX) subunit of the mitochondrial respiratory chain complex IV and participates in the association of complex III and IV. Knockdown of HIGD2A expression in HEK293 cells leads to dysregulated mitochondrial dynamics, decreased complex IV activity, and impaired complex IV-dependent respiration, and subsequently inhibits mitochondrial oxidative phosphorylation [19, 20]. Previously, higher expression of HIGD2A mRNA in HCC tissues was reported to be significantly associated with a poor prognosis [21]. However, little is known about the biological effects of elevated expression of HIGD2A in HCC.

The aim of this study was to investigate the role of HIGD2A in HCC development and to explore potential mechanisms. We conducted a comprehensive evaluation of the expression of HIGD2A in HCC and examined the association between the expression of HIGD2A and the prognosis of patients with HCC. Furthermore, to explore the role of HIGD2A in liver cancer biology, the effects of knocking down HIGD2A expression on cell proliferation, apoptosis, migration, and cell cycle were evaluated in vitro using HepG2, Huh7, and MHCC97H cell lines. Additionally, the function of HIGD2A in HCC progression was further validated in murine subcutaneous tumor xenograft models. Mechanistically, we explored the effects of HIGD2A knockdown on mitochondrial structure and function, as well as on the MAPK/ERK signaling pathway. Our findings demonstrated that elevated HIGD2A expression promotes the development of HCC by enhancing mitochondrial function and activating the MAPK/ERK pathway, which can be considered as a potential therapeutic target for HCC.

## Materials and methods

### Acquisition and processing of public HCC data

Datasets from The Cancer Genome Atlas (TCGA) and International Cancer Genome Consortium (ICGC) cohorts were downloaded (https://portal.gdc.cancer.gov/ and https://dcc.icgc.org/projects/LIRI-JP, respectively). The datasets of the GSE14520 and GSE64041 cohorts were downloaded from the GEO database (https://www.ncbi.nlm.nih.gov/geo/). Immunohistochemical (IHC) images of the HIGD2A protein in normal and tumor liver tissues were obtained from the Human Protein Atlas database (http://www.proteinatlas.org/) [22]. Survival analysis was performed using the R package survival. A nomogram combining the expression of HIGD2A and several clinical features was developed to predict the overall survival (OS) probability at 1, 2, and 3 years in the TCGA-LIHC cohort, and calibration curves and concordance indexes were used to test its validity. The Stemness index (mRNAsi) was calculated using the one-class logistic regression machine learning algorithm [23, 24]. A gene set enrichment analysis (GSEA) was performed using GSEA software (version 4.1.0) and the c2.all.v2022.1.Hs.symbols.gmt subset was downloaded from the portal (http://www.gsea-msigdb.org/gsea/index.jsp).

### Patients and specimens

Twenty-four patients with HCC who underwent tumor resection were randomly selected. Paired samples of HCC tissue and adjacent normal liver tissue were collected at the Nanfang Hospital of Southern Medical University (Guangdong, China) from 1 September 2019 to 1 February 2020. This study was approved by the Medical Ethics Committee of Nanfang Hospital of Southern Medical University, and written informed consent was obtained from participating patients.

### Cell culture, lentiviral infection and transfection

The human normal hepatic cell line L02 and the human HCC cell lines HepG2, Huh7, and MHCC97H were obtained from the Shanghai Cell Bank of the Academy of Chinese Sciences and Liver Cancer Institute, Zhongshan Hospital, Fudan University (China). All cell lines were cultured in complete Dulbecco’s Modified Eagle’s Medium (DMEM) containing 10% fetal bovine serum, 100 U/mL penicillin and 0.1 mg/mL streptomycin (Gibco, USA). All cells were kept in a humidified incubator at 37 °C with 5% CO_2_. The lentiviruses for shRNA-HIGD2A (shHIGD2A) and shRNA-scramble (shCtrl) were purchased from Shanghai Obio Technology Co. Ltd. (China). Lentiviral transduction was performed according to the manufacturer’s instructions. Cell transfection was performed using lipo3000 (Invitrogen, USA) as recommended by the manufacturer. PcDNA^TM^3.1-flag-BCL2L1 plasmid expressing human BCL2L1(GenBank No. NM_138578) was constructed by PCR from the respective cDNA clones using flag-tag encoding oligonucleotides followed by insertion into the pcDNA^TM^3.1 vector (Genechem, China).

### Western blotting analysis

Cell protein samples were lysed with loading buffer (7722, Cell Signaling Technology, USA) and were then heated at 100 °C for 10 min. Cell lysates were separated by sodium dodecyl sulfate-polyacrylamide gel electrophoresis (SDS-PAGE) and transferred to a PVDF membrane (Roche, USA) for immunoblotting with the following primary antibodies: anti-HIGD2A antibody (ab150893, Abcam, USA), anti-Mfn1 antibody (13798-1-AP, ProteinTech, USA), anti-OPA1 antibody (27733-1-AP, ProteinTech), anti-CD133 antibody (66666-1-Ig, ProteinTech), anti-EpCAM antibody (ab71916, Abcam), anti-JNK (9258, CST, USA), anti-pJNK (4668, CST), anti-Erk1/2 (4695, CST), anti-pErk1/2 (4377, CST), anti-P38 (9212, CST), anti-pP38 (9215, CST), anti-caspase3 (9662, CST), anti-cleaved caspase3 (9664, CST), anti-BCL2L1 (T40057F, Abmart, China) and anti-β-actin antibody (4970, CST) at 1:1000 dilution. The membranes were then incubated with secondary antibody for 1 h at room temperature. Finally, protein bands were visualized using ECL Prime Western Blot Detection Reagent (GE Healthcare, USA) and detected with ImageQuant LAS 4000mini (GE Healthcare).

### RNA extraction and quantitative real-time PCR

Total RNA was extracted using the EZ-press RNA Purification Kit (EZBioscience, USA) according to the manufacturer’s instructions. Real-time PCR was performed using the EZ-press One Step qRT-PCR Kit (EZBioscience) on the Roche 480 real-time PCR machine (Roche). β-Actin served as an internal control for real-time PCR. All primers used for real-time PCR are listed in Additional file 1: Table S1.

### Cell proliferation assay

A cell proliferation assay was conducted using the Cell Counting Kit-8 (CCK-8; Fude Biological Technology Co., China). After 48 h of lentivirus infection, HCC cells were seeded in 96-well plates (1000 cells/well) and incubated at 37 °C for 24, 36, 48, 72, and 96 h. Subsequently, the medium was added with 10 µL CCK-8 per well. The absorbance at 450 nm was measured after incubation for 2 h using Gen5 software (Biotek, USA).

### Colony formation assay

For the colony formation assay, after 48 h of cell infection, cells were seeded in a 6-well plate at a density of 2000 cells per well and incubated for 10 days. The cells were then fixed in 4% paraformaldehyde for 15 min, washed three times with PBS, and then stained with 1% crystal violet for 15 min. The number of colonies was counted using ImageJ software after staining the plates.

### Cell apoptosis and cell cycle assay

For the evaluation of cell apoptosis, lentivirus-infected cells were collected by trypsinization, centrifuged, and washed with PBS. The cells were then suspended in 200 µL binding buffer with 5 µL Annexin V and 5 µL 7-AAD (BioLegend, USA) for 15 min at room temperature. After staining, the percentage of apoptotic cells was analyzed using the FACS Canto™ II flow cytometer (BD Biosciences, USA).

To analyze the cell cycle, cells were harvested and fixed with 70% precooled ethanol at 4 °C overnight. After two washes, the fixed cells were incubated with 0.5 mL of PI/RNase staining buffer (BD Biosciences, USA) at room temperature for 15 min and then analyzed by flow cytometry.

### Cell migration assay

To explore the effects of HIGD2A on cell migration, 5 × 10^4^ infected cells were suspended in 100 µL of serum-free medium and then seeded in the upper chamber of an 8 µm-microporous filter (Corning, USA). The lower chamber of the Transwell was filled with 700 µL of medium supplemented with 20% FBS. After 48 h, cells that had not migrated were gently removed from the upper chambers and cells at the bottom of the chamber were fixed with polyformaldehyde for 15 min. The fixed cells were then stained with 1% crystal violet for 15 min. After two washes with PBS, the number of migrated cells was evaluated by microscope (IX73, Olympus, Japan) and captured images were evaluated with ImageJ software.

### Intracellular ATP level measurement

HepG2, Huh7 and MHCC97H cells were trypsinized and washed with PBS. A total of 1 × 10^6^ cells were used to detect intracellular ATP levels using the ATP Assay Kit (Beyotime, China) according to the manufacturer’s instructions.

### Immunofluorescence

A total of 5 × 10^4^ cells were seeded in a confocal microscopy dish and incubated for 24 h. The medium was removed from the plate and Opti-MEM containing 200 nM MitoTracker® Red CMXRos (M7512, Invitrogen, USA) was added. After incubation for 20 min, cells were washed twice with PBS and fixed in 4% paraformaldehyde for 20 min. Following three washes with PBS, the samples were counterstained with DAPI (Abcam, USA) for 5 min. Fluorescence was detected using a ZEISS LSM 880 confocal microscope with Airyscan (Carl Zeiss, USA). 3D rendering was performed with Imaris software (BitPlane, Zurich, Switzerland).

### Seahorse XF cell Mito Stress Test

Oxygen consumption was determined using a Seahorse XF cell Mito Stress Test kit (Agilent, USA) according to the manufacturer’s instructions. Briefly, HepG2, Huh7, and MHCC97H cells were infected with the lentivirus shHIGD2A or shCtrl and seeded onto Seahorse cell culture microplates at a density of 10,000, 7200, and 9000 cells/well, respectively. After more than 24 h, the oxygen consumption rate (OCR) was determined by the Seahorse XFe96 extracellular flux analyzer. Plates were analyzed in the presence of 1.5 µM oligomycin, 1.0 µM FCCP, or 0.5 µM rotenone/antimycin. Basal respiration and respiration capacity were measured.

### Seahorse XF glycolytic rate assay

The Seahorse XF Glycolytic Rate Assay kit was used to assay the glycolytic rate. HepG2 and Huh7 cells infected with lentivirus shHIGD2A or shCtrl were seeded as described for the Mito Stress Test. Rotenone/Antimycin at a concentration of 0.5 µM and 2-deoxy-d-glucose (2-DG) at 50 mM were used. The glycolytic proton efflux rate (GlycoPER) was calculated.

### RNA-sequencing

Total RNA was extracted from shCtrl- and shHIGD2A-infected HepG2 cells using a Trizol reagent kit (Invitrogen, USA) and libraries for RNA-sequencing were constructed. Differentially expressed genes (DEGs) was analyzed using DESeq2 software using the selection criteria of |log_2_Fold change| > 1 and a false discovery rate (FDR) < 0.05. Reactome enrichment analysis on DEGs was executed by ClusterProfiler software, and *P*-values < 0.05 were considered significantly enriched.

### Animal experiments

All animal study protocols were approved by the Animal Ethics Committee of the Nanfang Hospital of Southern Medical University (China). All male BALB/c-nu nude mice (3–5 weeks) were obtained from Hunan SJA Laboratory Animal Co., Ltd (China); animals were fed in a specific pathogen-free (SPF) vivarium under standard conditions. To construct the subcutaneous xenograft tumor model, HepG2, Huh7, and MHCC97H cells were infected with the lentivirus shHIGD2A or by a shCtrl. A 50-µL volume of cell suspension (5 × 10^6^ cells for each mouse) was injected into the right flank of each mouse. The growth of the xenografts was monitored at the indicated time points by measuring the tumor length (L) and width (W) to calculate tumor volume (V), using the following formula: V (mm^3^) = 0.5 × L × W^2^. On day 24 after inoculation, the mice were euthanized, and the excised tumors were weighed, photographed, and fixed in 4% paraformaldehyde overnight.

### Immunohistochemistry

After fixation in 4% paraformaldehyde overnight at room temperature, the excised tumor tissues were dehydrated, and embedded in paraffin. Tumor sections of 4 µm in size were stained with Ki67. After deparaffinization and rehydrating, heat-induced epitope repair was achieved by microwave boiling in the presence of sodium citrate buffer (10 mM sodium citrate, 0.05% Tween 20, pH 6.0) for 15 min. The sections were blocked with a PBS solution containing 5% BSA at room temperature for 1 h and incubated with Ki67 antibody (1:200, 9449, CST) at 4 °C overnight. Endogenous peroxidase activity was blocked with 3% hydrogen peroxide at 37 °C for 15 min. Then, an anti-rabbit/mouse IgG-HRP-linked secondary antibody (GK500710, Genetech, USA) was added for 1 h at room temperature. The sections were developed using 3-diaminobenzidine tetrahydrochloride at room temperature for about 2 min. Mayer’s hematoxylin was applied to stain the nucleus. Representative field photographs were captured using the BX63 microscope (Olympus) and were analyzed using ImageJ software.

### Statistical analysis

The Wilcoxon signed-rank test and Kruskal–Wallis test were performed to analyze the association between HIGD2A and clinical features in HCC. Kaplan–Meier analysis was used to compare the OS rate between the high and low HIGD2A gene expression groups using the p-value determined by the log-rank test. All statistical analyses were performed using R statistical software version 3.6.3 and 4.1.2. All data relative to the in vitro and in vivo experiments were analyzed using GraphPad 9.0. The Mann–Whitney U test or Student’s t-test were used to compare differences between the HIGD2A knockdown groups and the control group. Data normality was tested using a Shapiro–Wilk test. A p-value < 0.05 was considered statistically significant.

## Results

### HIGD2A was overexpressed and associated with a poor prognosis in patients with HCC

**Fig. 1:**
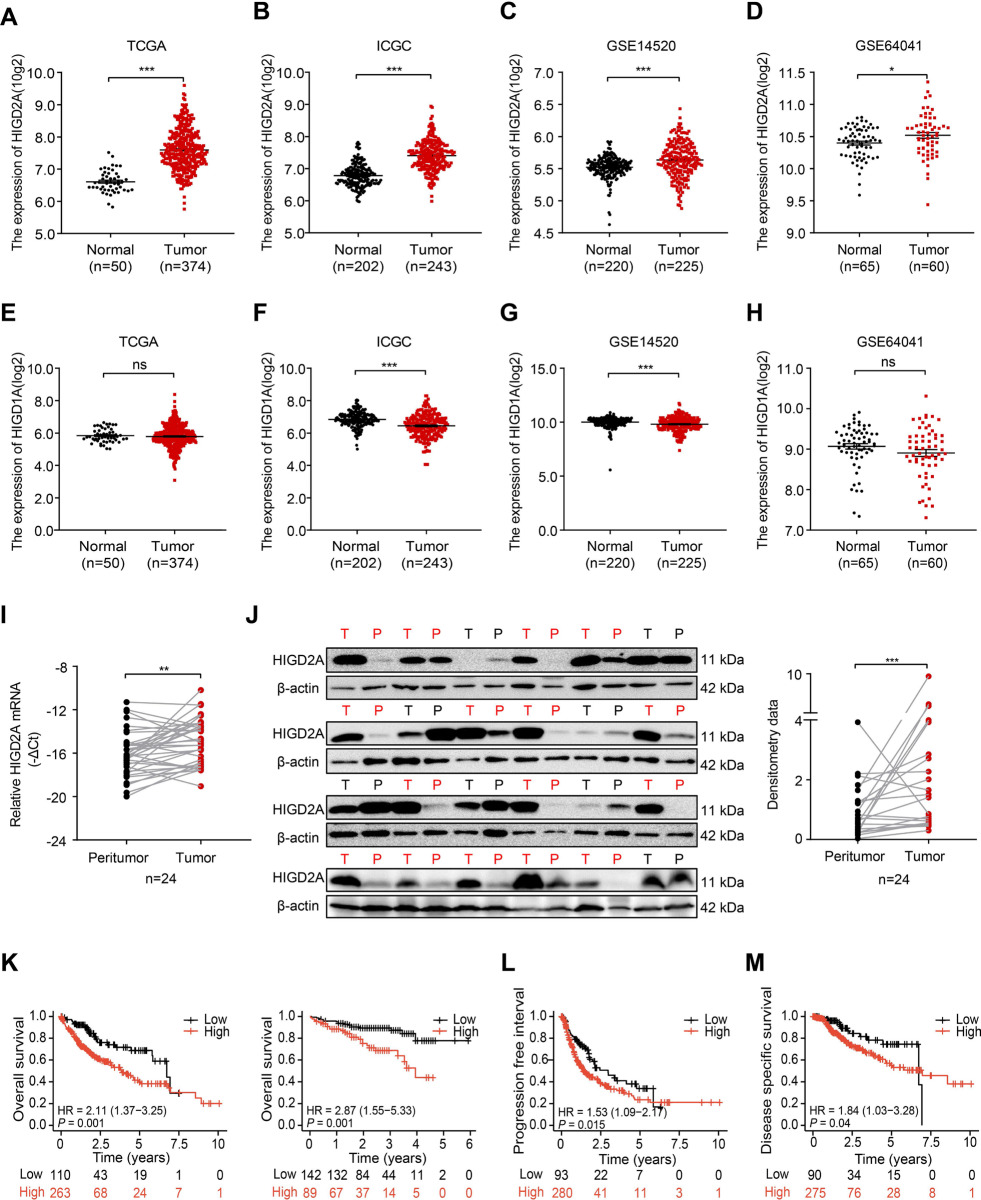
HIGD2A expression is elevated in HCC tissue and is correlated with poor prognosis. **A**–**H** Relative mRNA levels of HIGD2A and HIGD1A in normal and LIHC tissues from TCGA database (**A**, **E**), ICGC database (**B**, **F**), GSE14520 (**C**, **G**) and GSE64041 (**D**, **H**). LIHC, liver hepatocellular carcinoma. **I** HIGD2A mRNA levels in 24 pairs of HCC and matched peritumor tissues. **J** HIGD2A protein levels in 24 pairs of HCC and matched peritumor tissues were revealed by western blotting, and the bands were semi-quantified by densitometry and normalized to β-actin. Red indicates pairs with increased HIGD2A in cancer. *T* tumor, *P* peritumor. **K** Kaplan–Meier analysis of OS in HCC patients with high versus low HIGD2A mRNA expression in TCGA database (left) and ICGC database (right). **L**, **M** Kaplan–Meier analysis of PFI (**L**) and DSS (**M**) in patients with high versus low HIGD2A mRNA expression in TCGA database. Wilcoxon signed ranks test was used in **A**–**J**; log-rank tests were used in **K**–**M**. *P < 0.05, **P < 0.01 and ***P < 0.001; *ns* not significant

To investigate the potential significance of HIGD2A in the development and progression of HCC, we first analyzed multiple public databases, TCGA, ICGC, GSE14520, and GSE64041, and found that the expression of HIGD2A was significantly up-regulated in liver cancer tissues compared to the normal tissues (Fig. 1A–D). Immunohistochemical data for HIGD2A were obtained from the Human Protein Atlas (HPA) database and HIGD2A expression was also found to be up-regulated in HCC tissues (Additional file 1: Fig. S1A). To verify the results of the databases analyses, we examined the expression of HIGD2A in 24 pairs of HCC tissues and their matched pericancerous tissues. Consistent with previous results, the levels of HIGD2A mRNA (Fig. 1I) and protein (Fig. 1J) were significantly higher in HCC tissues than in their pericancerous counterparts. Furthermore, HIGD2A was universally expressed at higher levels in three HCC cell lines (HepG2, Huh7, and MHCC97H) with different oncogenic backgrounds compared to normal human hepatocyte L02 cells (Additional file 1: Fig. S1B). The above results indicate that HIGD2A expression is increased during HCC tumorigenesis.

Next, we evaluated the association of HIGD2A expression with clinical characteristics in patients with HCC. Analysis of the TCGA database and the ICGC database revealed that HCC patients with higher expression of HIGD2A had a shorter OS compared to patients expressing lower levels of HIGD2A (Fig. 1K). Higher expression of HIGD2A was associated with a worse progression-free interval (PFI) and disease-specific survival (DSS) (Fig. 1L, M). Furthermore, elevated expression of HIGD2A was significantly correlated with pathologic T stage, pathologic tumor stage, vascular invasion, and survival status (alive vs. dead) (Additional file 1: Fig. S1C–F). The subgroup analysis showed that a high expression of HIGD2A predominantly influenced the prognosis of patients with tumor stages T1, N0, and M0, and pathological stage (Additional file 1: Fig. S1G–J). A nomogram analysis of OS was subsequently performed using a multivariate Cox regression model (Additional file 1: Fig. S1K). By summing the points assigned to each variable and drawing a vertical line from the total point axis, the 1-, 3-, and 5-year OS times were predicted. An excellent agreement was observed between the predicted OS using the nomogram and the actual observed survival based on the calibration plots (Additional file 1: Fig. S1L). Taken together, our results indicate that HIGD2A was up-regulated in HCC tumor tissues, which was significantly associated with poorer survival in patients with HCC.

### Knockdown of HIGD2A inhibited HCC cells growth and migration

**Fig. 2:**
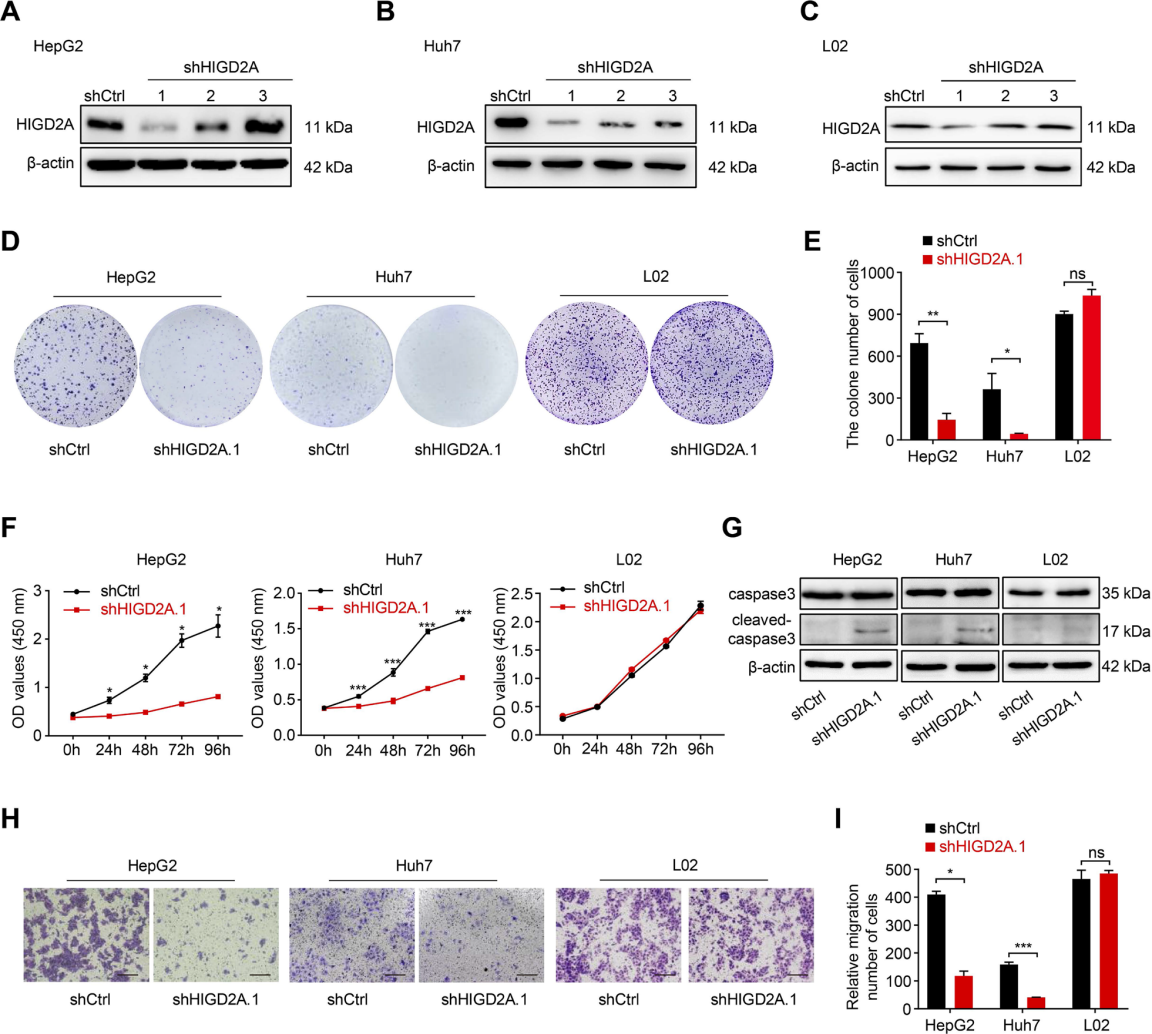
Silencing of HIGD2A impedes HCC cell proliferation and migration. **A**–**C** Western blotting assay for total HIGD2A protein expression following HIGD2A-knockdown in HepG2 (**A**), Huh7 (**B**) and L02 (**C**) cell lines. **D**, **E** Representative images of colony formation assay of HepG2, Huh7, and L02 cells transfected shCtrl or shHIGD2A.1 (**D**) and quantification of colony formation based on three independent assays (**E**). **F** The proliferation ability of HepG2, Huh7 and L02 cells infected with shCtrl or shHIGD2A.1 lentivirus was measured by CCK8 assay at the indicated time points. **G** The protein levels of caspase3 and cleaved-caspase3 were measured in HepG2, Huh7 and L02 cells infected with shCtrl or shHIGD2A.1 lentivirus. **H**, **I** Cell migration ability of HepG2, Huh7 and L02 cells transfected shCtrl or shHIGD2A.1 was analyzed by Transwell assay. Representative images of Transwell assay (**H**) and the quantitative data of migrated cells (**I**). Results shown are mean ± SEM. an unpaired t-test was used. *P < 0.05, **P < 0.01 and ***P < 0.001; ns, not significant. Scale bar, 100 µm

Because HIGD2A is highly expressed in three cancer cell lines, a lentivirus shRNA-mediated knockdown approach was used to identify the biological function of HIGD2A. The knockdown efficiency by different shRNAs was assessed using western blotting, which showed that shHIGD2A.1 exhibited the highest knockdown efficiency of HIGD2A (Fig. 2A–C and Additional file 1: Fig. S2A). Therefore, shHIGD2A.1 was chosen for our subsequent studies. Colony formation and CCK-8 assays were performed to examine the potential role of HIGD2A in the regulation of HCC cell proliferation. The colony formation assay revealed that HepG2, Huh7, and MHCC97H cells infected with shHIGD2A lentivirus had significantly fewer clones than the respective control cells (Fig. 2D, E, Additional file 1: Fig. S2B). Compared to control groups, HIGD2A knockdown significantly decreased cell proliferation in HepG2, Huh7, and MHCC97H cells (Fig. 2F, Additional file 1: Fig. S2C). Next, to determine whether growth arrest due to HIGD2A knockdown was associated with increased apoptosis, we examined the expression of apoptosis marker cleaved caspase3. Protein levels of cleaved caspase3 were obviously elevated in HIGD2A knockout cells (Fig. 2G). Furthermore, flow cytometric analyses also showed that the proportions of apoptotic cells (Annexin V+) increased markedly in HepG2, Huh7 and MHCC97H cell lines infected with shHIGD2A compared to the control groups (Additional file 1: Fig. S2D). These results indicated that the reduction in cell proliferation was due, at least in part, to apoptosis. The Transwell migration assay revealed that shRNA-mediated knockdown of HIGD2A attenuated the migratory ability of HepG2, Huh7 and MHCC97H cells (Fig. 2H, I, Additional file 1: Fig. S2G). Interestingly, HIGD2A knockdown had no effect on cell proliferation, apoptosis, and migration in the L02 cell line (Fig. 2D–I, Additional file 1: Fig. S2F).

To avoid off-target effects, shHIGD2A.2 was employed to confirm the results derived from shHIGD2A.1-mediated knockdown of HIGD2A because shHIGD2A.2 also showed remarkable knockdown efficiency (Fig. 2A, B). Consistent with the results reported above, HIGD2A knockdown in HepG2 and Huh7 cells markedly reduced cell proliferation, induced cell apoptosis, and decreased migration ability compared to controls (Additional file 1: Fig. S3A–G). Overall, these data demonstrated that HIGD2A played a role in HCC cell proliferation, apoptosis and migration.

### HIGD2A depletion inhibited cell proliferation in HCC cells by causing S phase cell cycle arrest

**Fig. 3:**
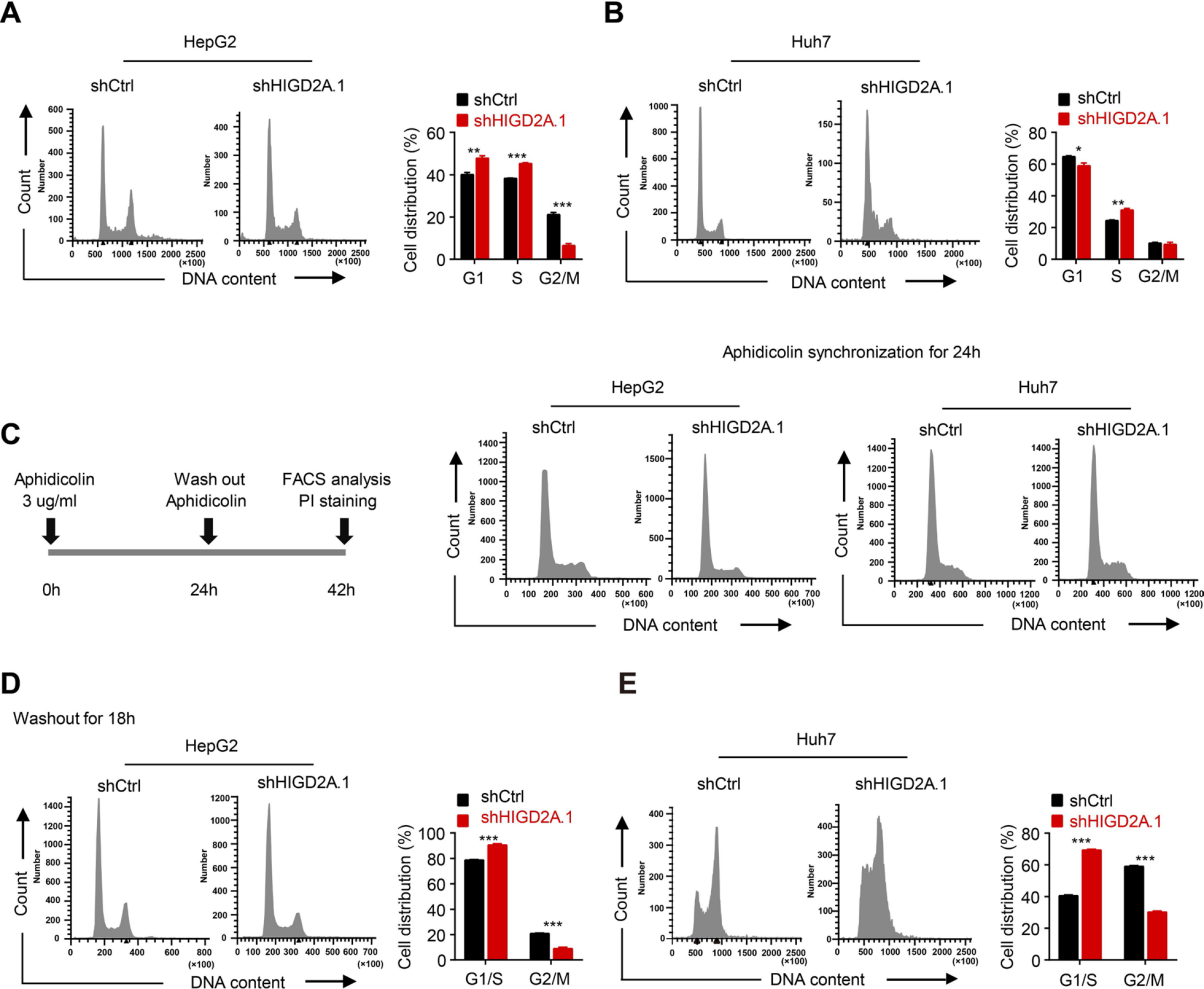
HIGD2A depletion induces cell cycle S phase arrest in HCC cells. **A**, **B** Cell cycle analysis of HepG2 (**A**) and Huh7 (**B**) cells treated with shCtrl or shHIGD2A.1 analyzed by FACS, and the proportion of cell population in G1, S, and G2/M phases. **C** Left, the timeline of the treatment with aphidicolin. Right, HepG2 and Huh7 treated with shCtrl or shHIGD2A.1 were synchronized with aphidicolin for 24 h and analyzed by FACS for the cell cycle. **D**, **E** 18 h after the removal of aphidicolin, HepG2 (**D**) and Huh7 (**E**) were stained with PI and analyzed by FACS. Right, the proportion of cell population in G1/S and G2/M phases after washout of Aphidicolin. Results shown are mean ± SEM. The Mann–Whitney test was used for statistical analyses. *P < 0.05, **P < 0.01 and ***P < 0.001

We determined that HIGD2A depletion significantly suppressed the growth of liver cancer cells in vitro. Thus, to further investigate the underlying mechanism of HIGD2A in the growth of HCC cells, we used propidium iodide (PI) staining to determine the effects of HIGD2A depletion on cell cycle distribution. Interestingly, silencing of HIGD2A resulted in an increase in the percentage of cells in the S phase compared to controls, suggesting that depletion of HIGD2A induced the arrest of the S phase in HepG2 and Huh7 cells (Fig. 3A, B). To further confirm this result, cancer cells were synchronized at the G1/S boundary using aphidicolin, a DNA polymerase inhibitor (Fig. 3C). After 24 h of synchronization of cell growth, aphidicolin was removed to initiate cell cycle progression, and PI staining was performed 18 h later. As expected, control cells transited into the G2/M phase, while more HIGD2A knockout cells remained stopped in the G1/S phase with no further progression (Fig. 3D, E). Taken together, these data indicated that HIGD2A knockdown delayed the proliferation and growth of HCC by inducing cell cycle arrest in the S phase.

### Depletion of HIGD2A induced mitochondrial stress and attenuated the ATP generation capacity of HCC cells

**Fig. 4:**
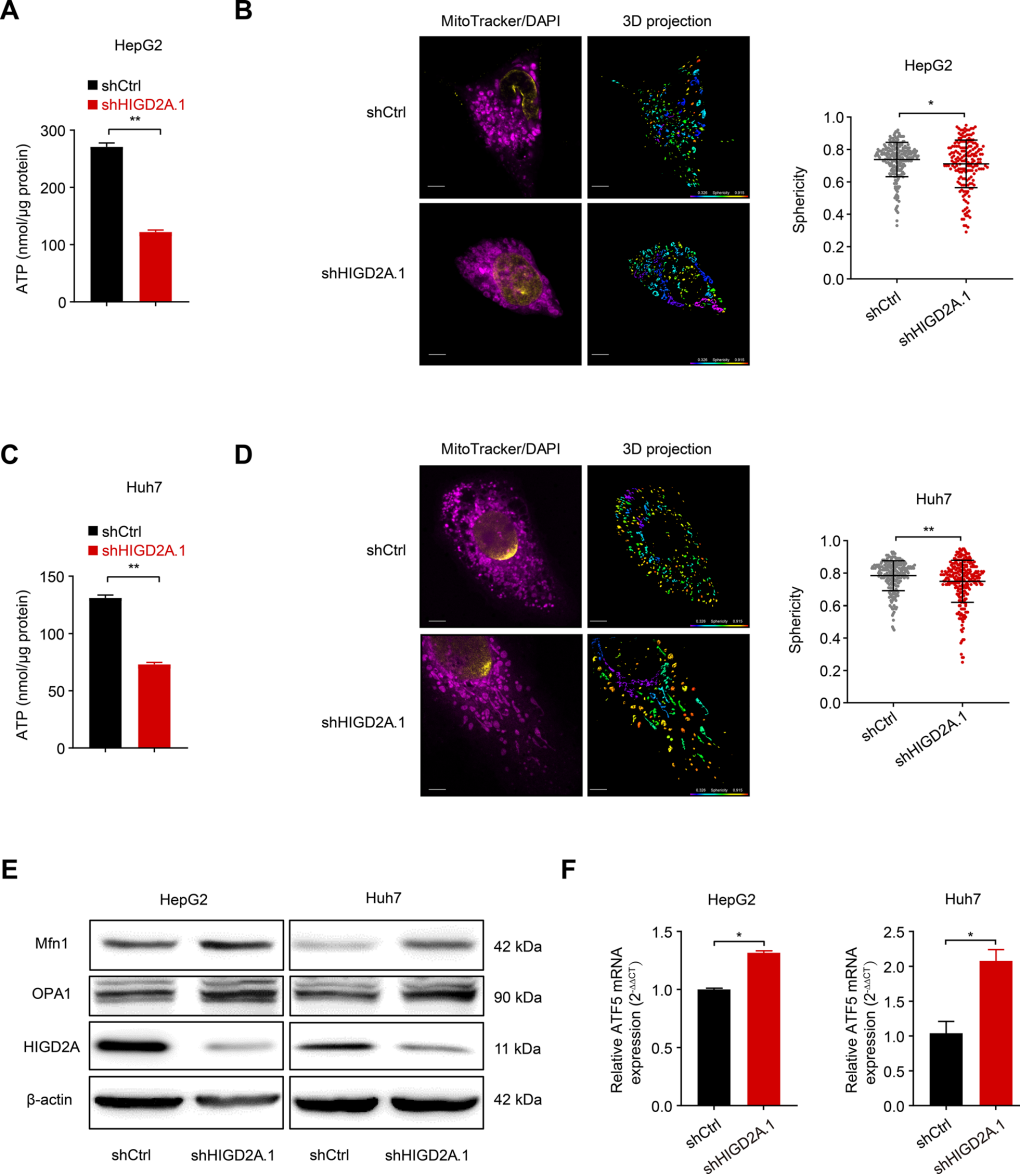
Depletion of HIGD2A induces mitochondrial stress. **A**, **C** Intercellular ATP level in HepG2 (**A**) and Huh7 (**C**) transfected with shCtrl or shHIGD2A.1. **B**, **D** Immunofluorescent (IF) images showing mitochondria morphology in HepG2 (**B**) and Huh7 (**D**) cells transfected with shCtrl or shHIGD2A.1. Left, representative IF images (magenta, MitoTracker; yellow, DAPI) and 3D mitochondria reconstruction of shCtrl- and shHIGD2A-infected HepG2 and Huh7 cell. Scale bar, 5 μm; Sphericity heat map, 0.326–0.915. Right, sphericity analysis of 3D reconstructed mitochondria (results are presented as mean ± SD). **E** Immunoblot analysis of mitochondrial fusion-related protein (Mfn1 and OPA1) in HepG2 (left) and Huh7 (right) transfected with shCtrl or shHIGD2A.1. **F** ATF5 mRNA level in in HepG2 (left) and Huh7 (right) transfected with shCtrl or shHIGD2A.1 measured by real-time PCR. Results shown are mean ± SEM. The Mann–Whitney test was used for statistical analyses. *P < 0.05 and **P < 0.01

Generally, the process of cell proliferation requires high consumption of ATP. Cancer cells often exhibit increased aerobic glycolysis to meet the metabolic demands of rapid cell proliferation and aggressiveness [25]. Therefore, we examined the impact of knocking down HIGD2A expression on cellular ATP levels. Knockdown of HIGD2A in HCC cells reduced ATP production compared to control groups (Fig. 4A, C and Additional file 1: Fig. S4A). In contrast, cellular ATP levels in L02 cells depleted with HIGD2A were comparable to those of control cells (Additional file 1: Fig. S4B). These data indicated that HIGD2A was essential for ATP generation in HCC cell lines.

Mitochondria are considered the 'powerhouse' of the cell, as they supply most of the cellular energy demand for ATP. Mitochondrial dynamics with repetitive fission and fusion cycles is important to ensure the maintenance of normal cell functions. For example, mitochondria are fused in the G1/S phase [26], and become increasingly fragmented in G2/M phase [27]. Mitochondrial fusion can also be triggered by energy depletion and stress conditions [10]. Given the impact of silencing HIGD2A expression on ATP production, we next investigated whether HIGD2A could regulate mitochondrial morphology. MitoTracker immunostaining was used to track mitochondria. Unexpectedly, a less round/elongated mitochondrial network was observed following HIGD2A knockdown in HCC cells (Fig. 4B, D, Additional file 1: Fig. S4C), which was consistent with our previous finding showing S phase arrest in HIGD2A-depleted cells. Furthermore, depletion of HIGD2A expression in HepG2 and Huh7 cells significantly increased the number of mitochondrial fusion-related proteins (MFN1, OPA1) (Fig. 4E). Furthermore, activating transcription factor 5 (ATF5), a mitochondrial stress response protein, was highly expressed in HIGD2A-depleted HCC cells (Fig. 4F), indicating that reduced expression of HIGD2A contributes to mitochondrial stress.

**Fig. 5:**
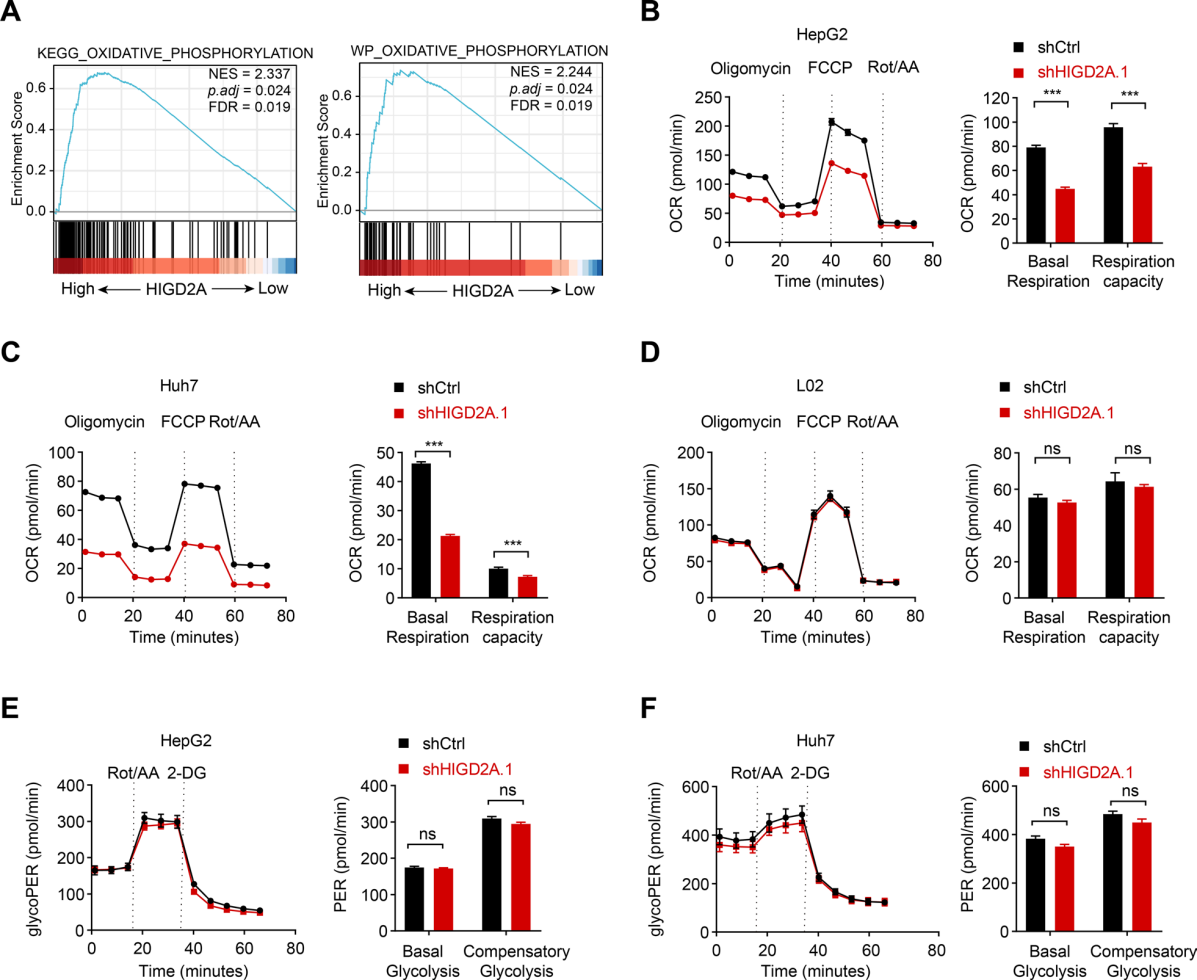
Knockdown of HIGD2A decreases mitochondrial oxidative phosphorylation. **A** Expression of genes associated with oxidative phosphorylation are significantly upregulated in patients with high HIGD2A mRNA expression. GSEA analysis of TCGA dataset (hepatocellular carcinoma). NES, normalized enrichment score. FDR, false discovery rate. **B**–**D** Left, oxygen consumption rate (OCR) in HepG2 (**B**), Huh7 (**C**) and L02 cells (**D**) transfected with shCtrl or shHIGD2A.1 were measured by seahorse analyzer. Right, basal respiration rate and maximal respiration capacity are shown. Rot/AA, rotenone/antimycin A. **E**–**F** The glycolytic proton efflux rate (glycoPER) in HepG2 (**E**) and Huh7 (**F**) transfected with shCtrl or shHIGD2A.1 was measured in an XFe96 analyzer after injection of Rot/AA and 2-deoxy-d-glucose (2-DG). Graphical analysis of Basal Glycolysis and Compensatory Glycolysis. Results shown are mean ± SEM. The Mann–Whitney test was used for statistical analyses. ***P < 0.001; *ns* not significant

Consistently, GSEA analysis of liver cancer samples from TCGA datasets indicated that genes related to oxidative phosphorylation were enriched in patients with HCC exhibiting greater expression of HIGD2A (Fig. 5A). To characterize the effects of HIGD2A depletion on mitochondrial oxidative phosphorylation function in more detail, the oxygen consumption rate (OCR) in HIGD2A knockdown cells was calculated. As expected, depletion of HIGD2A induced a significant decrease in the basal respiration rate and the maximum respiration capacity in the three HCC cell lines evaluated (Fig. 5B, C and Additional file 1: Fig. S4D). Consistent with our previous finding, knockdown of HIGD2A did not affect oxidative phosphorylation function in L02 cells (Fig. 5D). In parallel, the impact of HIGD2A on glycolytic function was also analyzed. As shown in Fig. 5E–F, HIGD2A knockdown had a limited effect on the glycolytic function in HepG2 and Huh7 cells. This result was not surprising given that HIGD2A contributes primarily to the assembly of the respiratory chain supercomplex. Furthermore, by comparing the basal respiration of shCtrl-infected HCC cells and L02 cells, we found that the basal respiration of HCC cells was higher than that of L02 cells (Additional file 1: Fig. S4E). This might be associated with a higher expression of HIGD2A in HCC cells. Collectively, these data demonstrate that HIGD2A is critical for the maintenance of mitochondrial morphology and mitochondrial oxidative phosphorylation function in HCC cells.

### HIGD2A might partially regulate the proliferation ability of HCC cells by modulating the activation of the MAPK/ERK signaling pathway

**Fig. 6:**
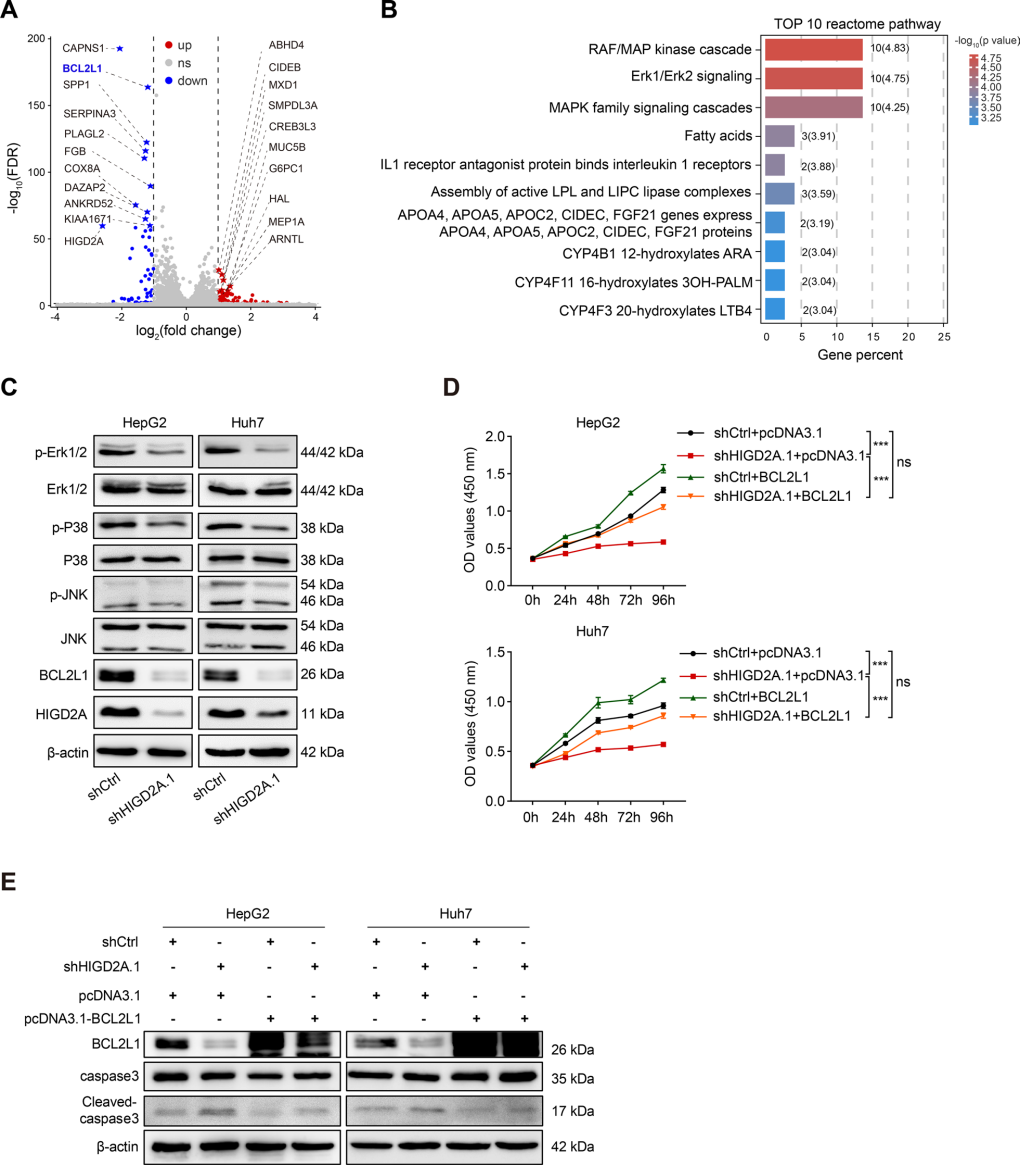
HIGD2A regulate proliferation ability of HCC cells partially by modulating MAPK/ERK signaling pathway. **A** Volcano plots of differential gene expression in shCtrl and shHIGD2A.1 infected HepG2 cells (data from 3 biological replicates). **B** Reactome enrichment analysis. Top 10 significantly enriched pathway in shHIGD2A-infected group were shown. **C** MAPK activation and the protein levels of BCL2L1 were assessed by western blotting in HepG2 and Huh7 cells transfected with shCtrl or shHIGD2A.1 lentivirus. **D**, **E** BCL2L1 overexpression alleviated HIGD2A knockdown-mediated growth inhibition and apoptosis in HCC cells measured by CCK8 assay (**D**) and western blotting (**E**). Results shown are mean ± SEM. Two-way ANOVA with Tukey multiple comparison test was used for statistical analyses. ***P < 0.001; *ns* not significant

To uncover the molecular mechanisms underlying HIGD2A regulation of HCC proliferation, we performed RNA sequencing on shCtrl- and shHIGD2A.1-infected HepG2 cells. A total of 124 genes (66 up- and 58 down-regulated genes) exhibited significantly altered mRNA expression in shHIGD2A.1-infected cells, and the top 10 most significantly up-regulated and 10 down-regulated genes were selected (Fig. 6A). The enrichment pathways of 124 differential genes were analyzed through the Reactome pathway database, which revealed that the RAF/MAP kinase cascade and Erk1/Erk2 signaling pathways were the most significantly enriched (Fig. 6B). Given the importance of the MAPK/ERK pathway in HCC cell proliferation, migration and apoptosis [28], we next examined MAPK/ERK pathways activation in HIGD2A knockdown cells. Silencing of HIGD2A markedly decreased phosphorylation of Erk1/2 and suppressed the MAPK/ERK pathway in HCC cells (Fig. 6C). Interestingly, other MAPK pathways, including JNK and P38 MAPK cascades, were also suppressed by HIGD2A knockdown (Fig. 6C). These data suggested that HIGD2A knockdown-mediated tumor suppression might be relevant by blocking of the MAPK/ERK pathway.

We then investigated how the MAPK/ERK pathway influenced the growth of HIGD2A-depleted HCC cells. Among the most aberrantly expressed genes identified by RNA-sequencing, we found that BCL2L1, an anti-apoptotic gene whose expression is induced by activation of the MAPK/ERK pathway [29], was significantly down-regulated. Consistently, the protein levels of BCL2L1 were also decreased in shHIGD2A.1-infected cells (Fig. 6C). Because BCL2L1 plays an important role in tumor cell survival and proliferation [30], we next investigated whether overexpression of BCL2L1 could restore tumor growth in shHIGD2A.1-infected cells. As expected, BCL2L1 overexpression reversed HIGD2A knockdown-mediated growth inhibition and apoptosis in HepG2 and Huh7 cells (Fig. 6D, E). Overall, these results indicated that silencing of HIGD2A inhibited tumor cell proliferation and survival partially by blocking the MAPK/ERK pathway and repressing the expression of BCL2L1.

### HIGD2A promoted the stemness properties of hepatocellular carcinoma

**Fig. 7:**
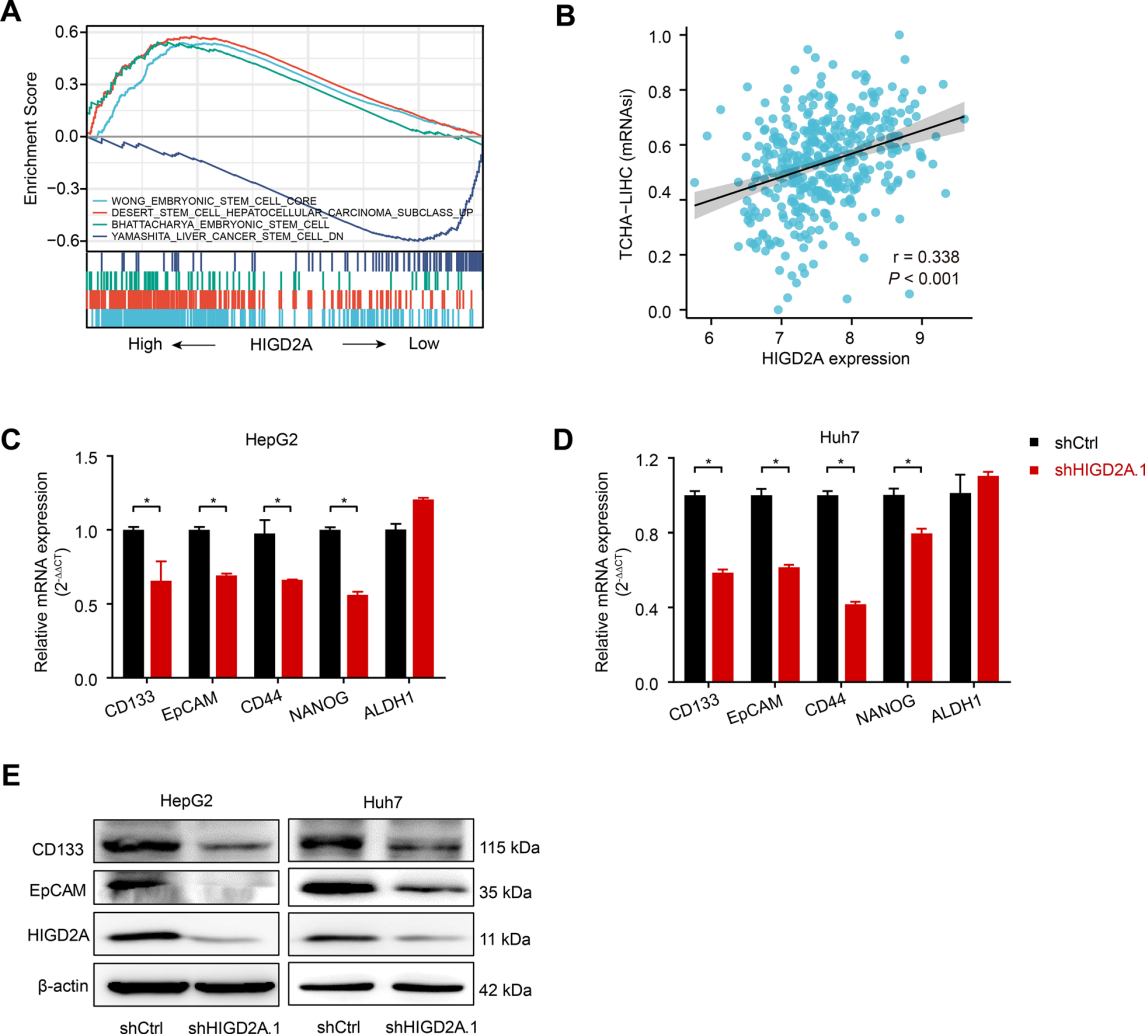
HIGD2A promotes stemness property of hepatocellular carcinoma. **A** Expression of genes associated with tumor stemness are significantly upregulated in patients with high HIGD2A mRNA expression. GSEA analysis of TCGA dataset (hepatocellular carcinoma). **B** Positive association of mRNAsi with HIGD2A expression. **C**, **D** The mRNA expression levels of the tumor stemness-associated markers (CD133, EpCAM, CD44, NANOG, ALDH1) in HepG2 (**C**) and Huh7 (**D**) transfected with shCtrl or shHIGD2A.1 were measured by real-time PCR. **E** Immunoblot analysis of the protein levels of CD133 and EpCAM in HepG2 and Huh7 transfected with shCtrl or shHIGD2A.1. Results shown are mean ± SEM. Pearson correlation was used in B and The Mann–Whitney test was used in C-D for statistical analyses. *P < 0.05

A recent study showed that liver cancer stem cells (LCSCs) undergo enhanced glycolysis and oxidative phosphorylation (OXPHOS) compared to nonstem cells, and reducing OXPHOS levels weakens the stemness properties of LCSCs [31]. Given the role of HIGD2A in regulating mitochondrial OXPHOS, we next explored the relationship between HIGD2A expression and tumor stemness of HCC cells. As shown in Fig. 7A, signaling pathways related to tumor stemness were enriched in patients with HCC with high expression of HIGD2A compared to those with low HIGD2A expression. Particularly, mRNAsi, a stemness index used to assess the dedifferentiation potential of tumor cells, was also positively associated with the expression of HIGD2A (Fig. 7B). These findings suggested that HIGD2A promoted tumor stemness in HCC. To further confirm these results, the effects of HIGD2A knockdown on the expression of CSC markers were examined. The expression of liver CSC markers (including CD133, EpCAM, CD44, NANOG, but not ALDH1) was significantly reduced after silencing of HIGD2A (Fig. 7C, D). A similar decrease in CD133 and EpCAM protein levels was also observed in the knockdown group (Fig. 7E). Taken together, our data indicate that HIGD2A contributes to the maintenance of the stemness properties of HCC.

### Silencing of HIGD2A expression interfered with the tumorigenicity of HCC cell lines in xenografted nude mice

**Fig. 8:**
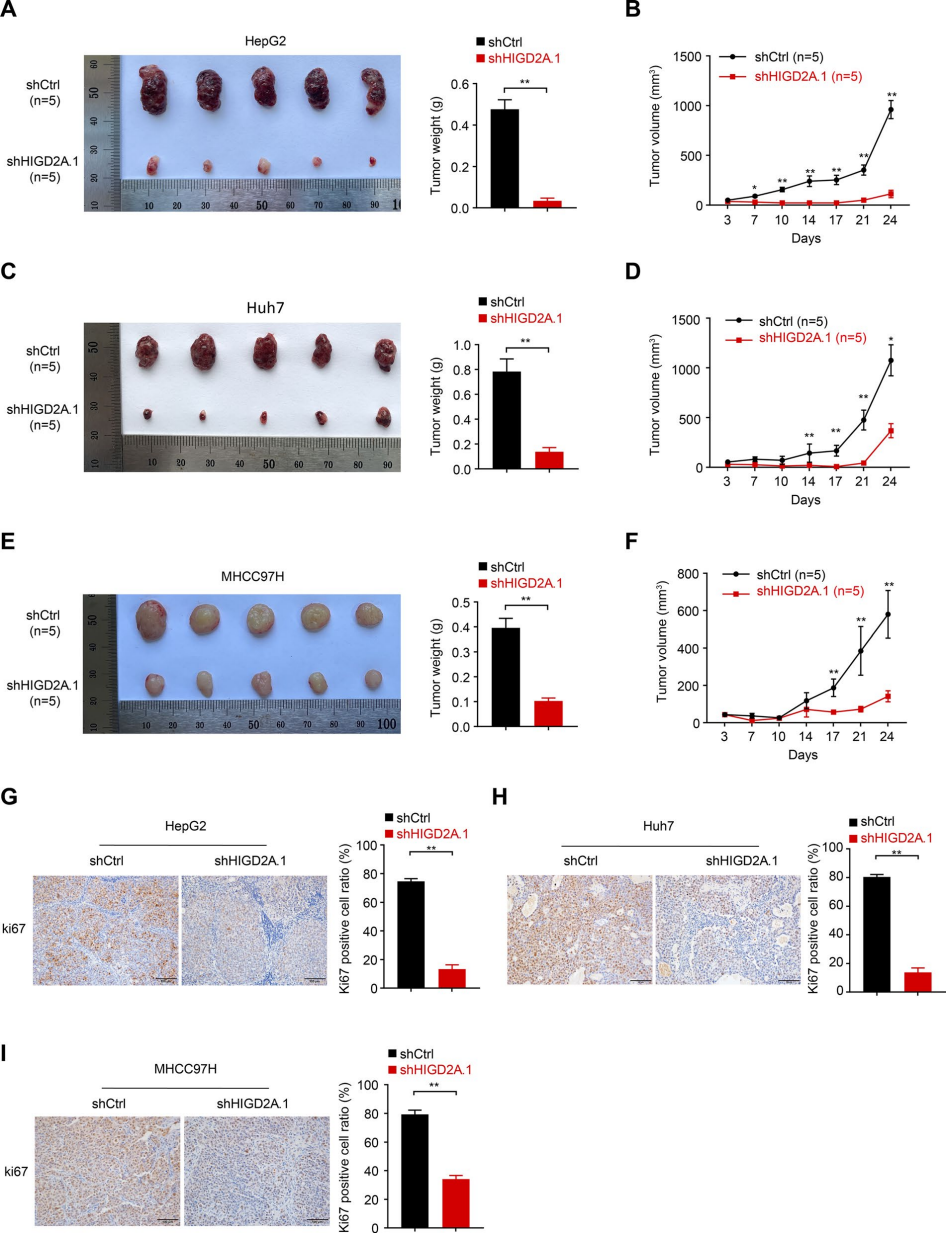
Knockdown of HIGD2A attenuated the tumorigenicity of HCC cell lines in vivo. **A**–**F** Images of HepG2 (**A**), Huh7 (**C**) and MHCC97H (**E**) xenograft tumors in nude mice of shCtrl group and shHIGD2A.1 group (n = 5 mice per group), and comparison of the tumor weight between shCtrl group and shHIGD2A.1 group. Tumor volume changes of HepG2 (**B**), Huh7 (**D**) and MHCC97H (**F**) were monitored at indicated time points. **G**–**I** Immunohistochemical staining of ki67 in HepG2 (**G**), Huh7 (**H**) and MHCC97H (**I**) xenograft tumor tissues from shCtrl group and shHIGD2A.1 group, and the proportion of ki67 positive cells counted by ImageJ software. Results shown are mean ± SEM. **A** Mann–Whitney test was used. *P < 0.05 and **P < 0.01. Scale bar, 100 µm

Given the growth retardation phenotype detected in HIGD2A knockdown cells, we subsequently sought to determine whether silencing of HIGD2A expression influenced the proliferation of HepG2, Huh7 and MHCC97H xenografted tumors in nude mice. Consistent with the in vitro results, knock-down expression of HIGD2A in HepG2, Huh7 and MHCC977H xenografted tumors resulted in growth retardation and in significantly reduced in tumor volumes and weights compared to control tumors (Fig. 8A–F). Meanwhile, IHC staining revealed that Ki67 expression, a marker of cell proliferation, was markedly decreased in tumor tissues harboring HIGD2A knockdown compared to control tissues (Fig. 8G–I). Collectively, our results further suggested that HIGD2A promotes tumorigenicity of HCC cells in the mouse model.

## Discussion

**Fig. 9:**
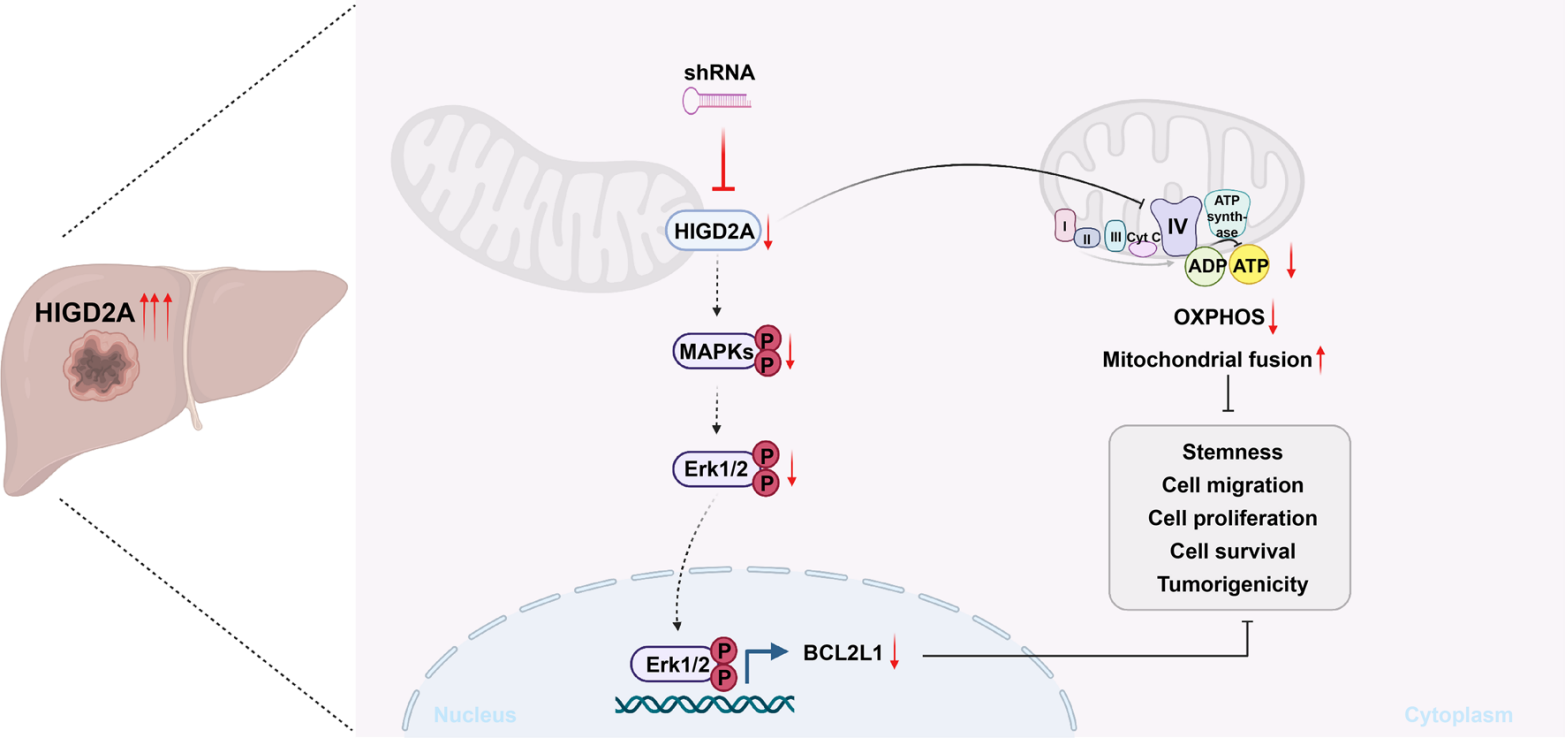
A model describing knockdown of HIGD2A attenuates hepatocellular carcinoma growth by causing mitochondrial dysfunction and the suppression of MAPK/ERK pathway. Image generated with BioRender

In this study, we detected an increased expression of HIGD2A in patients with liver cancer, and these higher levels of HIGD2A were associated with a poor prognosis. Knockdown of HIGD2A expression delayed in vitro cellular proliferation and caused cell cycle arrest in the S phase of HCC cells, which was associated with compromised mitochondrial function and reduced ATP production. Furthermore, HIGD2A knock-down inhibited the activation of the MAPK/ERK pathway and the expression of BCL2L1, which could promote liver cancer cell apoptosis and suppress cell proliferation (Fig. 9). Thus, we proposed that HIGD2A could act as a potential prognostic marker and therapeutic target for HCC.

The respiratory supercomplex factor Rcf1 has two mammalian homologues, HIGD1A and HIGD2A [20]. HIGD1A is up-regulated in a variety of human cancers and has been implicated as an oncogene because it promotes cell proliferation and survival [32–34]. However, in our study, the expression of HIGD1A in HCC was comparable to or even slightly lower than that detected in normal tissue samples obtained from public datasets (Fig. 1E–H), although HIGD2A expression was markedly elevated in patients with liver cancer. Furthermore, we showed that HIGD2A was essential for the proliferation of HCC cells, while HIGD2A depletion had a limited influence on cell growth in normal hepatocyte L02 cells. The most probable reason is that lower expression of HIGD2A observed in normal hepatocytes may be dispensable for normal cell growth and HIGD1A can partially substitute for the function of HIGD2A [20]. This observation was consistent with previous studies reporting that HIGD2A expression was associated with different cell proliferation rates in mouse tissue [19] and HIGD2A depletion selectively impaired the viability of colon adenocarcinoma cells [35]. Analysis of TCGA database revealed that HIGD2A expression was upregulated and associated with a poor prognosis in various types of human cancers, not just in patients with HCC [21]. Future studies should explore the role of HIGD2A in the development of other tumors.

Mitochondrial dynamics is tightly regulated by fusion and fission proteins [36]. Recent studies have shown that mitochondrial fission is positively correlated with the metastatic capacity of HCC cells [12]. In the HIGD2A knockout HEK293 cell line, a significant increase in the expression of OPA1 protein involved in mitochondrial fusion was observed, while FIS1, another mitochondrial fission protein, showed a significantly lower expression [19]. In this study, a hyperfused mitochondrial network and higher levels of mitochondrial fusion-related proteins (MFN1, OPA1) were identified in HIGD2A-knockdown HCC cells. This result confirmed that HIGD2A was involved in the regulation of mitochondrial dynamics. Moreover, HIGD2A knockdown weakened the migration ability of HCC cells, and the expression of HIGD2A was also much higher in patients with HCC exhibiting vascular invasion.

Given the improved understanding of the deregulation of cancer metabolism, targeting the mitochondria may act as a potentially powerful cancer therapy [4, 37]. The development of inhibitors of complexes in the respiratory chain is an example of mitochondrial-targeted therapy. For example, metformin, an inhibitor of the electron transport chain complex 1, has been evaluated as tumor treatment [38]. HIGD2A is necessary for the generation of COX3, a subunit that participates in the modulation of complex IV activity [39, 40]. Knockdown of HIGD2A expression attenuates complex IV enzymatic activity in HEK293T cells [20, 41]. In our study, we demonstrated that HIGD2A knockdown decreased mitochondrial ATP production, mediated mitochondrial stress, led to cell cycle arrest in the S phase, and inhibited the proliferation of HCC cells. These results provide a strong rationale for exploring whether targeting HIGD2A or other molecules involved the mitochondrial metabolic pathway may represent therapeutic strategies for the treatment of HCC.

CSCs have the capacity for self-renewal and differentiation and contribute to tumorigenesis, tumor recurrence, metastasis, and therapeutic resistance [42]. In HCC, CSCs participate in therapeutic resistance and are associated with a poorer response to therapy with sorafenib [43]. Interestingly, our results indicated that HIGD2A was an important contributor to stemness maintenance of HCC cells, as the elimination of HIGD2A attenuated the expression of tumor stemness markers; however, additional functional experiments are required in the future to clarify the underlying mechanism.

There were some limitations in this study that should be considered. The first is that HIGD2A is not only highly expressed in tumor tissues but is also strongly expressed in highly proliferative tissues [19], which poses a great challenge for developing HIGD2A-targeted treatment strategy for HCC. Second, although knockdown of HIGD2A had profound effects on the cancer phenotype in HCC cells, it would be more convincing if the same results were obtained by overexpression of HIGD2A. Third, modulation of mitochondrial function by HIGD2A was confirmed in three HCC cell lines, these results would be more convincing if also validated in additional HCC cell lines. Finally, as HIGD2A protein is located mainly in the mitochondrial inner membrane, it is unknown how the MAPK/ERK signaling pathway is influenced by HIGD2A. Further studies are required to elucidate the role of HIGD2A in the MAPK/ERK pathway in HCC.

## Conclusions

Our study determined that knockdown of HIGD2A suppressed the proliferation and growth of HCC cells by interfering with the MAPK/ERK pathway and suggested that HIGD2A may play a role in abnormal mitochondrial metabolism in HCC. The present study provides novel evidence for the role of HIGD2A in hepatocarcinogenesis and may also have potential significance as a therapeutic target. Further studies should elucidate the precise mechanism of HIGD2A activity in the modulation of the MAPK/ERK pathway, and explore the role of HIGD2A in HCC metastasis, and as a feasible to target for liver cancer therapy.

## Supplementary Information

**Additional file 1: Table S1.** The shRNA sequences of HIGD2A and the primer sequences used in real-time PCR analysis. **Figure S1.** Diagnostic and prognostic value of HIGD2A expression and its correlation with clinical features. **A** Representative immunohistochemistry images of HIGD2A expression in normal liver tissues and liver cancer tissues from the HPA. **B** The protein level of HIGD2A in normal hepatocyte L02 cell line and different liver cancer cell lines. **C**–**F** HIGD2A expression in different status of T stage (**C**), pathologic stage (**D**), vascular invasion (**E**) and OS event (**F**). **G**–**J** Kaplan–Meier plots of OS for HIGD2A expression levels in subgroups including T stage: T1 (**G**), N stage: N0 (**H**), M stage: M0 (**I**) and pathologic stage: stage I (**J**). **K** Nomogram for OS prediction, with T stage, N stage, M stage, histologic grade and expression of HIGD2A applied as parameters. **L** Calibration curves of the nomogram for 1-, 3-, 5-year survival prediction. *P < 0.05, **P < 0.01. **Figure S2.** Knockdown of HIGD2A impedes MHCC97H cells proliferation and migration. **A** Western blot assay for total HIGD2A protein expression in HIGD2A-knockdown MHCC97H cells. **B** Left, colony formation of MHCC97H cells transfected shCtrl or shHIGD2A.1. Right, quantification of colony formation based on three independent assays. **C** The proliferation ability of MHCC97H cells infected with shCtrl lentivirus or shHIGD2A.1 lentivirus was measured by CCK8 assay at the indicated time points. **D**, **E** Flow cytometry analysis of Annexin V/7-AAD double stained HepG2, Huh7 and MHCC97H cells transfected shCtrl or shHIGD2A.1. Representative flow cytometric plot (**D**) and the proportion of apoptosis cells (**E**). **F** Annexin V/7-AAD apoptosis assay of L02 cells after HIGD2A gene silencing. Left, representative flow cytometric plot**.** Right, the proportion of apoptosis cells. **G** Left, cell migration ability of MHCC97H cells transfected shCtrl or shHIGD2A.1 was analyzed by Transwell assay. Right, quantitative data of migrated cells. Results shown are mean ± SEM. an unpaired t-test was used. *P < 0.05, **P < 0.01, ***P < 0.001, ns, not significant. Scale bar, 100 µm. **Figure S3.** HIGD2A knockdown inhibited the proliferation and migration of HCC cells in vitro. **A**, **B** Colony formation experiments for the effect of HIGD2A knockdown with shRNA on the proliferation of HepG2 and Huh7 cells. **C**, **D** The effect of HIGD2A knockdown on the growth of HepG2 and Huh7 cells was detected by CCK8 assays. **E** The effect of HIGD2A knockdown on cell apoptosis was detected by western blot. **F**, **G** Transwell chamber was used to evaluated the effect of HIGD2A knockdown on the migration of HepG2 and Huh7 cells. Results shown are mean ± SEM. an unpaired t-test was used. *P < 0.05, **P < 0.01 and ***P < 0.001. Scale bar, 100 µm. **Figure S4.** Depletion of HIGD2A induces mitochondrial stress in MHCC97H. **A**, **B** Intercellular ATP level in MHCC97H (**A**) and L02 (**B**) cells transfected with shCtrl or shHIGD2A.1. **C** Immunofluorescent (IF) images showing mitochondria morphology in MHCC97H transfected with shCtrl or shHIGD2A.1. Left, representative IF images (magenta, MitoTracker; yellow, DAPI) and 3D mitochondria reconstruction of shCtrl- and shHIGD2A-infected MHCC97H cell. Scale bar, 5 μm; Sphericity heat map, 0.326–0.915. Right, sphericity analysis of 3D reconstructed mitochondria (results are presented as mean ± SD). **D** Left, oxygen consumption rate (OCR) in MHCC97H transfected with shCtrl or shHIGD2A.1 was measured by seahorse analyzer. Right, basal respiration rate and maximal respiration capacity are shown. **E** Comparison of Basal Respiration among L02 and HCC cells. Results shown are mean ± SEM. A Mann–Whitney test was used. *P < 0.05, **P < 0.01 and ***P < 0.001; ns, not significant.

## Data Availability

All data generated or analyzed during this study are included in the manuscript and Additional file.

## Abbreviations

HIGD2A: Hypoxia Inducible Gene Domain family member 2A
HCC: Hepatocellular carcinoma
ATP: Adenosine triphosphate
ROS: Reactive oxygen species
TCGA: The Cancer Genome Atlas
ICGC: International Cancer Genome Consortium
HPA: Human Protein Atlas
OS: Overall survival
PFI: Progression-free interval
DSS: Disease-specific survival
OCR: Oxygen consumption rate
ATF5: Activating transcription factor 5
OXPHOS: Oxidative phosphorylation
LCSCs: Liver cancer stem cells
