# Supplementary material for: HIGD2A silencing impairs hepatocellular carcinoma growth via inhibiting mitochondrial function and the MAPK/ERK pathway
Source: J Transl Med. 2023 Apr 12;21:253. doi: 10.1186/s12967-023-04105-7 (PMC10091548; doi:10.1186/s12967-023-04105-7)
Supplement: Supplementary file 1 — Additional file 1: Table S1. The shRNA sequences of HIGD2A and the primer sequences used in real-time PCR analysis. Figure S1. Diagnostic and prognostic value of HIGD2A expression and its correlation with clinical features. A Representative immunohistochemistry images of HIGD2A expression in normal liver tissues and liver cancer tissues from the HPA. B The protein level of HIGD2A in normal hepatocyte L02 cell line and different liver cancer cell lines. C–F HIGD2A expression in different status of T stage (C), pathologic stage (D), vascular invasion (E) and OS event (F). G–J Kaplan–Meier plots of OS for HIGD2A expression levels in subgroups including T stage: T1 (G), N stage: N0 (H), M stage: M0 (I) and pathologic stage: stage I (J). K Nomogram for OS prediction, with T stage, N stage, M stage, histologic grade and expression of HIGD2A applied as parameters. L Calibration curves of the nomogram for 1-, 3-, 5-year survival prediction. *P < 0.05, **P < 0.01. Figure S2. Knockdown of HIGD2A impedes MHCC97H cells proliferation and migration. A Western blot assay for total HIGD2A protein expression in HIGD2A-knockdown MHCC97H cells. B Left, colony formation of MHCC97H cells transfected shCtrl or shHIGD2A.1. Right, quantification of colony formation based on three independent assays. C The proliferation ability of MHCC97H cells infected with shCtrl lentivirus or shHIGD2A.1 lentivirus was measured by CCK8 assay at the indicated time points. D, E Flow cytometry analysis of Annexin V/7-AAD double stained HepG2, Huh7 and MHCC97H cells transfected shCtrl or shHIGD2A.1. Representative flow cytometric plot (D) and the proportion of apoptosis cells (E). F Annexin V/7-AAD apoptosis assay of L02 cells after HIGD2A gene silencing. Left, representative flow cytometric plot. Right, the proportion of apoptosis cells. G Left, cell migration ability of MHCC97H cells transfected shCtrl or shHIGD2A.1 was analyzed by Transwell assay. Right, quantitative data of migrated cells. Results shown a [file 12967_2023_4105_MOESM1_ESM.docx]

Additional file 1

**HIGD2A silencing impairs hepatocellular carcinoma growth via inhibiting mitochondrial function and the MAPK/ERK pathway**

Kuiyuan Huang^#, 1^, Ziying Liu^#, 1^, Zhanglian Xie^#, 1^, Xiaoran Li^1^, Haixing Zhang^1^, Yu Chen^1^, Yiran Wang^1^, Zimo Lin^1^, Chuanjiang Li^🖂, 2^, Hongyan Liu^🖂, 1^, Xiaoyong Zhang^🖂, 1^

^1^ State Key Laboratory of Organ Failure Research, Guangdong Provincial Key Laboratory of Viral Hepatitis Research, Department of Infectious Diseases, Nanfang Hospital, Southern Medical University, Guangzhou, China;

^2^ Division of Hepatobiliopancreatic Surgery, Department of General Surgery, Nanfang Hospital, Southern Medical University, Guangzhou, China.

Xiaoyong Zhang, Email: [xiaoyzhang@smu.edu.cn](mailto:xiaoyzhang@smu.edu.cn)

Contributor Information.

^🖂^Corresponding author.

^#^Contributed equally.

| ShHIGD2A.1 | ACCATCGAAGCCTCCAGTCAT |
| --- | --- |
| shHIGD2A.2 | CAGGAATCCAGAGAGTTTCAA |
| shHIGD2A.3 | CTATGAAGTCTCGACCCTAAG |
| shCtrl | CCTAAGGTTAAGTCGCCCTCG |
| *HIGD2A* Forward | AGGAAAAGTTCGTTCGCAAG |
| *HIGD2A* Reverse | CTGAGAGCGCTGGCTGTT |
| *CD133* Forward | TTCTTGACCGACTGAGACCCA |
| *CD133* Reverse | TCATGTTCTCCAACGCCTCTT |
| *EpCAM* Forward | TGTGGTTGTGGTGATAGCAGTT |
| *EpCAM* Reverse | CCCATCTCCTTTATCTCAGCCTTC |
| *CD44* Forward | AAGGTGGAGCAAACACAACC |
| *CD44* Reverse | ACTGCAATGCAAACTGCAAG |
| *NANGO* Forward | CCAACATCCTGAACCTCAGCTAC |
| *NANGO* Reverse | GCCTTCTGCGTCACACCATT |
| *ALDH1* Forward | TGTTAGCTGATGCCGACTTG |
| *ALDH1* Reverse | TTCTTAGCCCGCTCAACACT |
| *ATF5* Forward | AGGGGACCGCAAGCAAAAG |
| *ATF5* Reverse | GCCTTGTAAACCTCGATGAGC |
| *Actin* Forward | ATCACCATTGGCAATGAGCG |
| *Actin* Reverse | TTGAAGGTAGTTTCGTGGAT |

**Table S1**


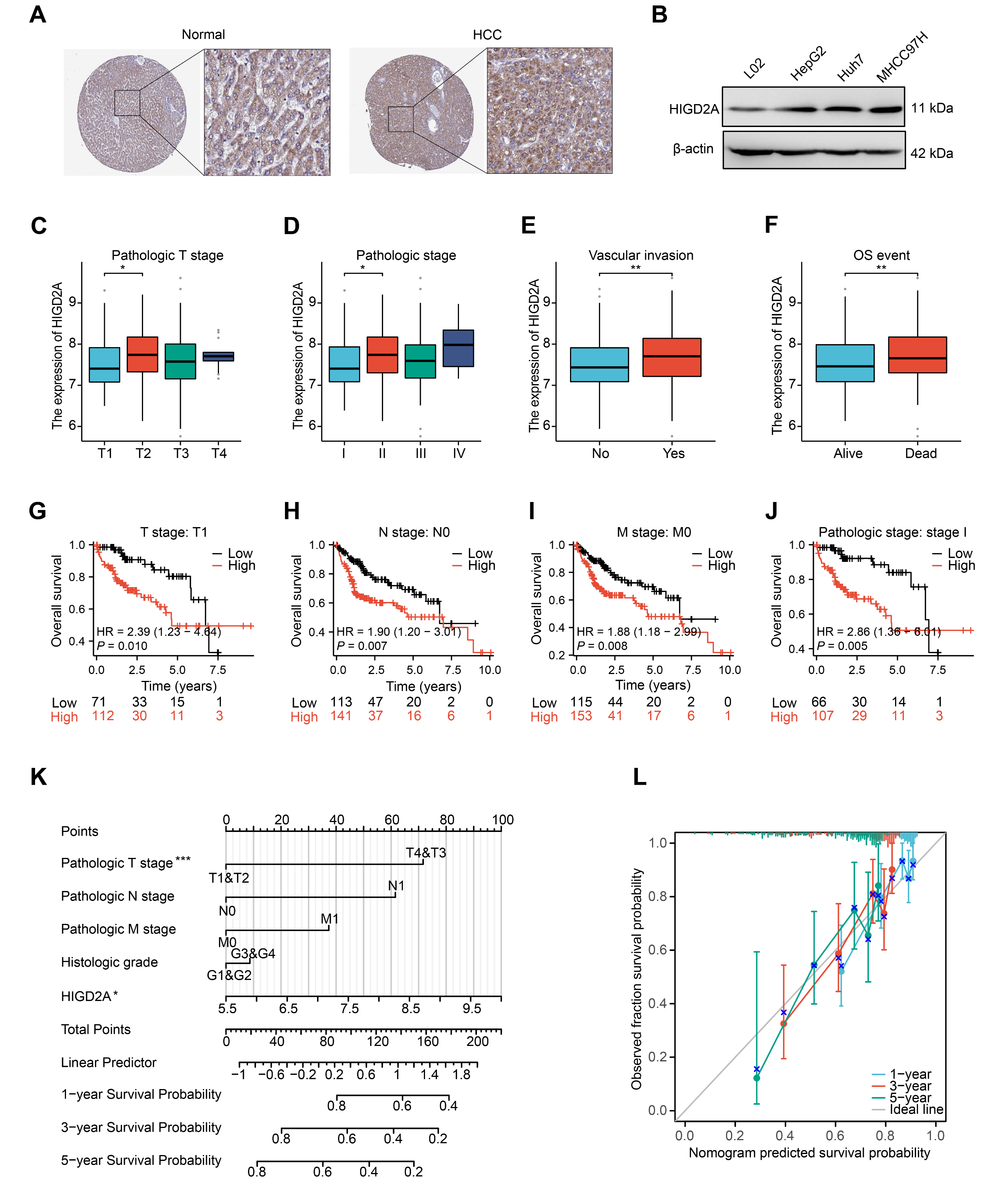


**Fig. S1**

Diagnostic and prognostic value of HIGD2A expression and its correlation with clinical features. **(A)** Representative immunohistochemistry images of HIGD2A expression in normal liver tissues and liver cancer tissues from the HPA. **(B)** The protein level of HIGD2A in normal hepatocyte L02 cell line and different liver cancer cell lines. **(C**–**F)** HIGD2A expression in different status of T stage **(C)**, pathologic stage **(D)**, vascular invasion **(E)** and OS event **(F)**. **(G–J)** Kaplan-Meier plots of OS for HIGD2A expression levels in subgroups including T stage: T1 **(G)**, N stage: N0 **(H)**, M stage: M0 **(I)** and pathologic stage: stage I **(J)**. **(K)** Nomogram for OS prediction, with T stage, N stage, M stage, histologic grade and expression of HIGD2A applied as parameters. **(L)** Calibration curves of the nomogram for 1-, 3-, 5-year survival prediction. *^*^P* < 0.05, *^**^P* < 0.01.


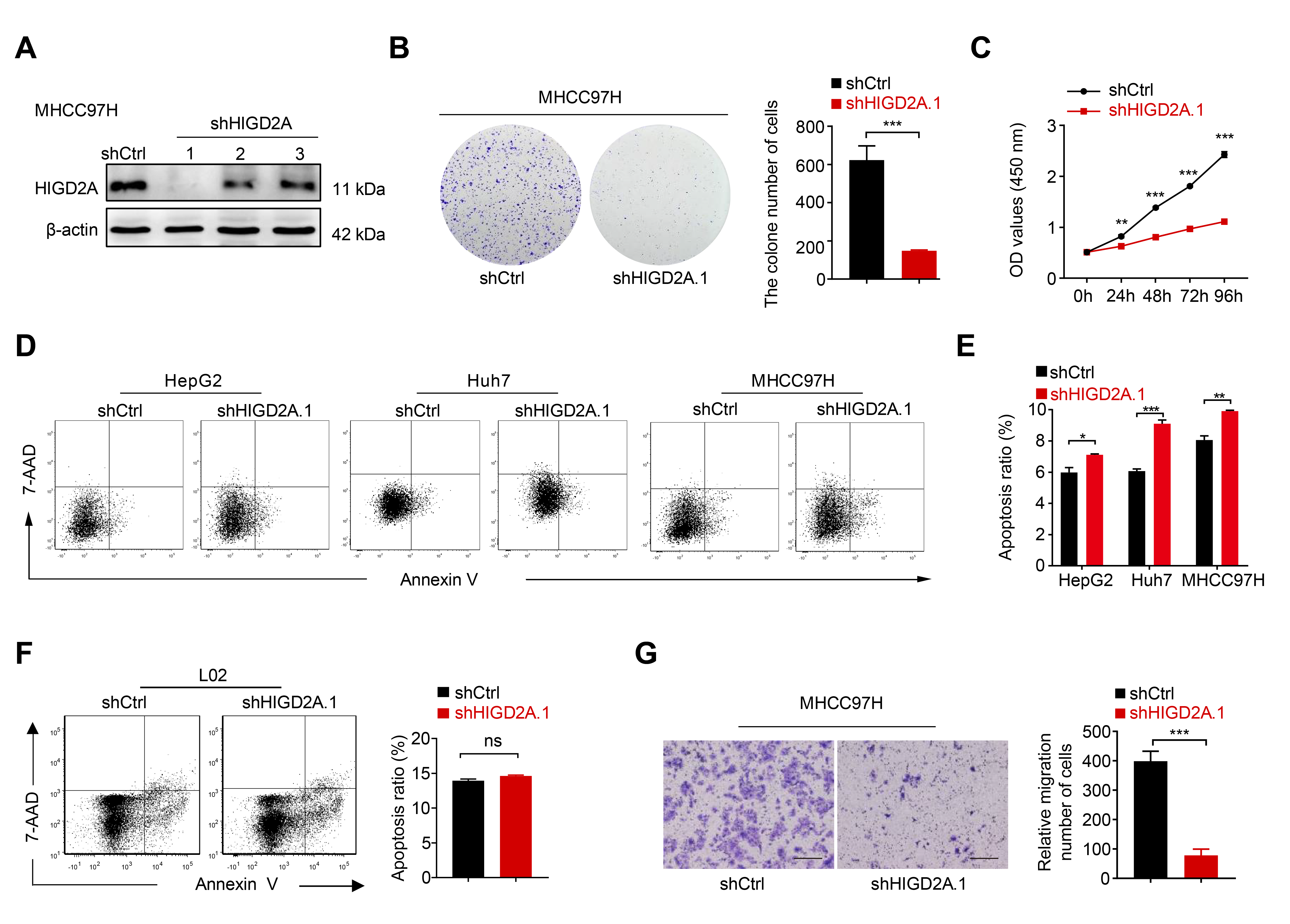


**Fig. S2**

Knockdown of HIGD2A impedes MHCC97H cells proliferation and migration. **(A)** Western blot assay for total HIGD2A protein expression in HIGD2A-knockdown MHCC97H cells. **(B)** Left, colony formation of MHCC97H cells transfected shCtrl or shHIGD2A.1. Right, quantification of colony formation based on three independent assays. **(C)** The proliferation ability of MHCC97H cells infected with shCtrl lentivirus or shHIGD2A.1 lentivirus was measured by CCK8 assay at the indicated time points. **(D, E)** Flow cytometry analysis of Annexin V/7-AAD double stained HepG2, Huh7 and MHCC97H cells transfected shCtrl or shHIGD2A.1. Representative flow cytometric plot **(D)** and the proportion of apoptosis cells **(E)**. **(F)** Annexin V/7-AAD apoptosis assay of L02 cells after HIGD2A gene silencing. Left, representative flow cytometric plot**.** Right, the proportion of apoptosis cells. **(G)** Left, cell migration ability of MHCC97H cells transfected shCtrl or shHIGD2A.1 was analyzed by Transwell assay. Right, quantitative data of migrated cells. Results shown are mean ± SEM. an unpaired t-test was used. *^*^P* < 0.05, *^**^P* < 0.01, *^***^P* < 0.001, ns, not significant. Scale bar, 100µm.

**
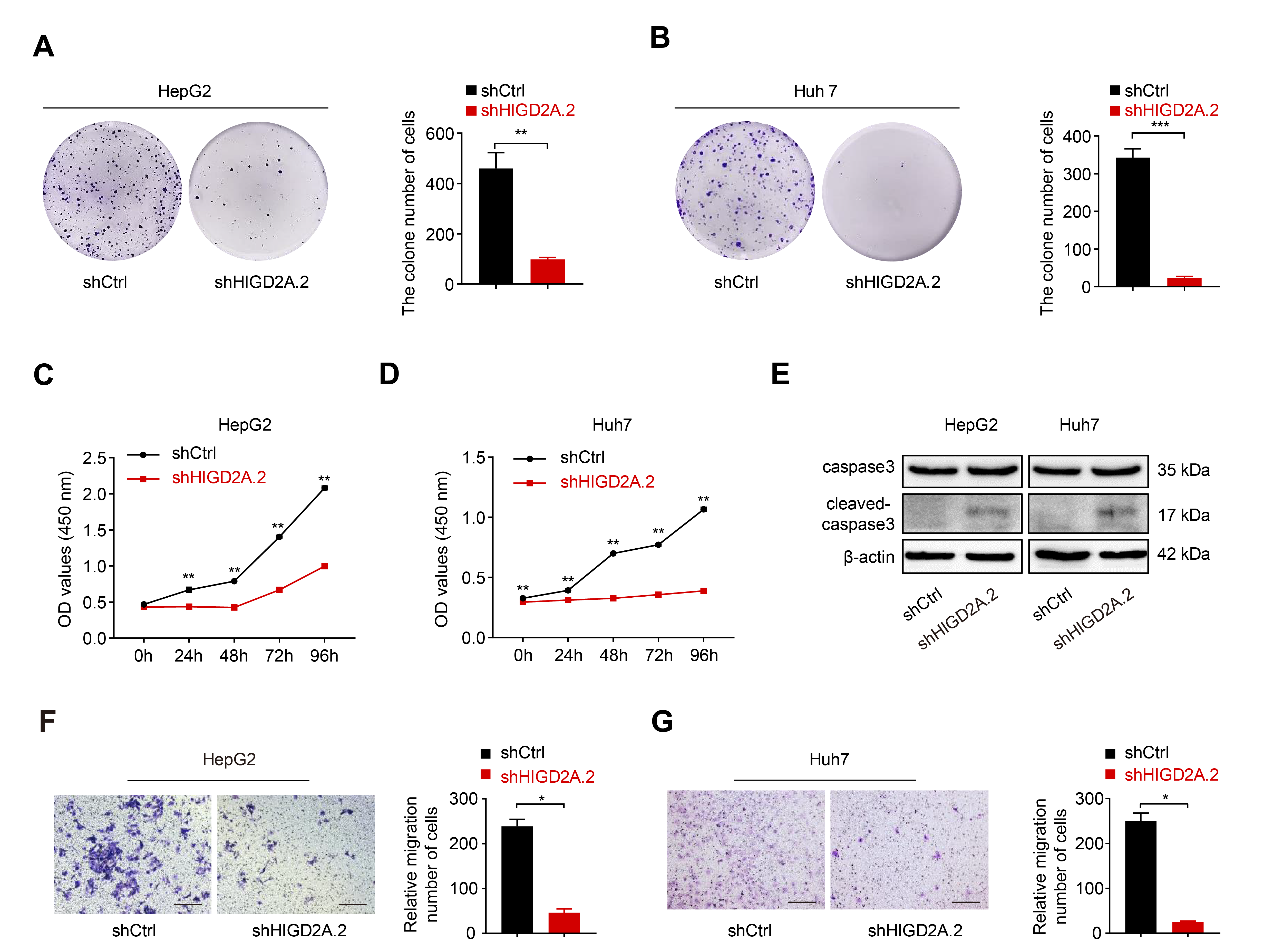
**

**Fig. S3**

HIGD2A knockdown inhibited the proliferation and migration of HCC cells in vitro. **(A, B)** Colony formation experiments for the effect of HIGD2A knockdown with shRNA on the proliferation of HepG2 and Huh7 cells. **(C, D)** The effect of HIGD2A knockdown on the growth of HepG2 and Huh7 cells was detected by CCK8 assays. **(E)** The effect of HIGD2A knockdown on cell apoptosis was detected by western blot. **(F, G)** Transwell chamber was used to evaluated the effect of HIGD2A knockdown on the migration of HepG2 and Huh7 cells. Results shown are mean ± SEM. an unpaired t-test was used. *^*^P* < 0.05, *^**^P* < 0.01 and *^***^P* < 0.001. Scale bar, 100µm.





**Fig. S4**

Depletion of HIGD2A induces mitochondrial stress in MHCC97H. **(A, B)** Intercellular ATP level in MHCC97H **(A)** and L02 **(B)** cells transfected with shCtrl or shHIGD2A.1. **(C)** Immunofluorescent (IF) images showing mitochondria morphology in MHCC97H transfected with shCtrl or shHIGD2A.1. Left, representative IF images (magenta, MitoTracker; yellow, DAPI) and 3D mitochondria reconstruction of shCtrl- and shHIGD2A-infected MHCC97H cell. Scale bar, 5μm; Sphericity heat map, 0.326-0.915. Right, sphericity analysis of 3D reconstructed mitochondria (results are presented as mean ± SD). **(D)** Left, oxygen consumption rate (OCR) in MHCC97H transfected with shCtrl or shHIGD2A.1 was measured by seahorse analyzer. Right, basal respiration rate and maximal respiration capacity are shown. **(E)** Comparison of Basal Respiration among L02 and HCC cells. Results shown are mean ± SEM. A Mann-Whitney test was used. *^*^P* < 0.05, *^**^P* < 0.01 and *^***^P* < 0.001; ns, not significant.
